# Dexmedetomidine post-conditioning protects blood-brain barrier integrity by modulating microglia/macrophage polarization *via* inhibiting NF-κB signaling pathway in intracerebral hemorrhage

**DOI:** 10.3389/fnmol.2022.977941

**Published:** 2022-09-08

**Authors:** Hao Guo, Weiwei Zhang, Zhi Wang, Zhishan Li, Jing Zhou, Zhaoyu Yang

**Affiliations:** ^1^Department of Anesthesiology, Shanxi Provincial People’s Hospital, Affiliate of Shanxi Medical University, Taiyuan, China; ^2^The First Central Clinical School, Tianjin Medical University, Tianjin, China; ^3^Shanxi Province Academy of Traditional Chinese Medicine, Shanxi Province Hospital of Traditional Chinese Medicine, Taiyuan, China; ^4^Department of Integrated Traditional Chinese and Western Medicine, Xiangya Hospital, Institute of Integrative Medicine, Central South University, Changsha, China; ^5^National Clinical Research Center for Geriatric Disorders, Xiangya Hospital, Central South University, Changsha, China

**Keywords:** intracerebral hemorrhage, dexmedetomidine, blood-brain barrier, microglia/macrophage polarization, NF-κB signaling pathway

## Abstract

Intracerebral hemorrhage (ICH) is one of the most devastating forms of stroke. Dexmedetomidine (DEX) has shown certain neuroprotective roles in ICH. Nevertheless, the details concerning the underlying molecular mechanism of DEX’s protective effects still need further elucidation. Herein, a model of ICH was established. The rats were randomly divided into the sham group, the ICH group, and the ICH + DEX group. Neurological outcomes, neuronal injury, and apoptosis were evaluated. Brain water content, Evans blue extravasation, and the expression of tight junction-associated proteins were also detected to assess the blood-brain barrier (BBB) integrity. Subsequently, the microglia/macrophage polarization state and inflammatory cytokine levels were observed. To further explore the underlying mechanism, NF-κB signaling pathway-associated proteins were detected. The results showed that DEX exerted neuroprotective effects against ICH-induced neurological deficits. DEX significantly increased the numbers of the surviving neurons and ameliorated neuronal cell loss and apoptosis in ICH. The rats that received the DEX displayed a lower level of brain water content and EB extravasation, moreover, ZO-1, occludin, and claudin-5 were markedly increased by DEX. Additionally, DEX facilitated M2 microglia/macrophage polarization, the M1-associated markers were reduced by DEX, while the M2-associated identification significantly increased. We found that DEX dramatically diminished pro-inflammatory cytokines expression, simultaneously promoting anti-inflammatory cytokines expression. DEX inhibited nuclear translocation of NF-κB in ICH rats. Our data suggest that DEX post-conditioning protects BBB integrity by modulating microglia/macrophage polarization *via* inhibiting the NF-κB signaling pathway in ICH.

## Graphical Abstract

DEX post-conditioning protects BBB integrity by modulating microglia/macrophage polarization via inhibiting NF-κB signaling pathway in ICH. 
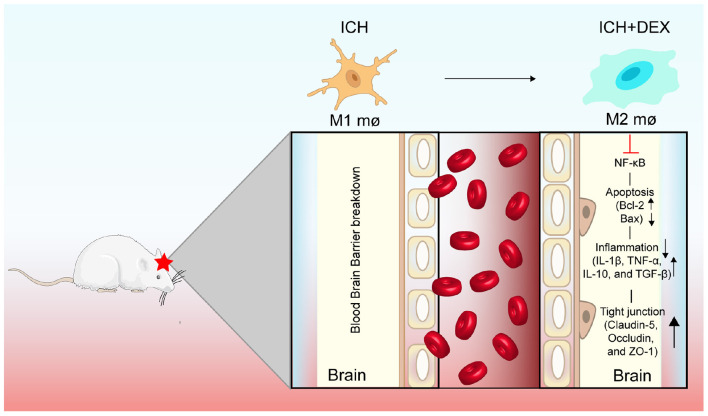

## Introduction

Intracerebral hemorrhage (ICH), one of the most devastating forms of stroke, has caused severe concern for public health because of its high morbidity and mortality (Baharoglu et al., 2016; Hanley et al., 2017). ICH accounts for approximately 15% of strokes. However, the 28-day mortality for ICH is disproportionately up to 47%. And among these patients who survived at 28 days, 44% had recurrent ICH at 5 years (Chen et al., 2020a). The neurological deficits of the ICH survivors will cause a substantial burden and reduce their quality of life. More disappointingly, despite extensive research and allocation of resources, advances in medical treatments have not obtained satisfactory results. Exploring effective therapies for ICH is still urgently needed.

Dexmedetomidine (DEX) is a highly selective adrenergic α2 receptor agonist. It has been widely used in clinical anesthesia and intensive care. Increasing evidence suggests that DEX exerts neuroprotective roles in brain injury. It can reduce the release of pro-inflammatory factors and prevent neutrophil infiltration after stroke (Yin et al., 2018). Moreover, post-conditioning with DEX ameliorates subarachnoid hemorrhage-induced brain injury by activating the extracellular signal-regulated kinase (Wang et al., 2016). Clinical studies have shown that DEX administration for patients with ICH during the perioperative period has specific brain protective effects (Zhao and Zhou, 2016). In the experimental model of ICH, DEX can effectively inhibit the activation of NLRP3 (Song and Zhang, 2019) and inhibit mitochondrial dysfunction-derived oxidative stress (Huang and Jiang, 2019). Nevertheless, based on the complicated pathological mechanisms of ICH, the details concerning the underlying molecular mechanism of DEX’s protective effects in ICH still need further elucidation.

The blood-brain barrier (BBB) breakdown leads to brain edema and hemorrhagic transformation, which plays a non-negligible role in ICH (Keep et al., 2014; Li et al., 2018). Reducing the BBB damage after ICH is neuroprotective and beneficial (Zhao et al., 2020). Accumulating evidence has indicated that reactive microglia/macrophages likely contribute to the leaky BBB, inflammation, oxidative stress, and immune cell trafficking in ICH (Haruwaka et al., 2019; Kang et al., 2020). Once activated, microglia/macrophages in the resting state (M0) can be phenotypically polarized into either the M1 phenotype or M2 phenotype (Xiong et al., 2016; Al Mamun et al., 2020). M1 microglia/macrophages release pro-inflammatory cytokines (such as TNF-α, IL-1β, and IL-6), which are typically assumed to promote brain damage (Higashi et al., 2017; Yu et al., 2019). M2 microglia/macrophages produce anti-inflammatory cytokines (IL-10, IL-4, and TGF-β), contributing to tissue repair and remodeling (Akhtar et al., 2017; Calvello et al., 2017). Hence, promoting the shift from the M1 phenotype toward the M2 phenotype to maintain the integrity of BBB may serve as a new promising therapeutic approach in ICH (Chen et al., 2020b).

In the current study, we aim to clarify the neuroprotective pharmacological effects and illustrate the underlying mechanism of DEX by post-conditioning the ICH model of rats. The results indicate that DEX does have protective effects against ICH-induced brain injury, these effects are related to the NF-κB signaling pathway-mediated microglia/macrophage M2 polarization.

## Materials and methods

### Experimental intracerebral hemorrhage model

Male Sprague-Dawley (SD) rats (200∼250 g) were obtained from the SPF (Beijing) Biotechnology Co., Ltd. The animals were housed with free access to food and water under a 12-h light/dark cycle. The ICH model was induced according to the previous method (Zhou et al., 2018). Briefly, rats were anesthetized (50 mg/kg pentobarbital sodium) and were positioned on a stereotaxic frame (Stoelting Co., Chicago, IL, United States). Then, 2.5 μL of 0.9% sterile saline with 0.5 U collagenase VII was injected into the right basal ganglia (Coordinate: the 1.4 mm posterior, 3.2 mm lateral to the bregma, and 5.6 mm ventral to the cortical surface). The injection lasted for over 2 min, with the needle kept in position for an additional 5 min. 2.5 μL of 0.9% sterile saline without collagenase was injected into the sham group.

### Experimental groups and administration of drugs

All rats were randomly divided into the sham group, the ICH group, and the ICH + DEX group. The ICH and ICH + DEX group underwent the ICH procedure. The animals in the DEX-treated group received the dose of DEX with 20 μg/kg/d intraperitoneally once a day for consecutive 1, 3, or 7 days (Heng Rui Medicine Co., Ltd., China), while the sham and ICH groups were administered with the same volume of vehicle.

### Neurobehavioral test

Two investigators who were blinded to the experimental groups evaluated the modified neurological severity score (mNSS), corner turn test, and foot-fault test. On 1st, 3rd, and 7th days post-ICH, the two investigators scored the tests independently; then, the scores were averaged. The mNSS was an integrative test to evaluate the motor, sensory, and reflex abilities. And a higher score meant a more severe injury. The corner turn test could assess the motor function. Rats were made to proceed into a corner with an angle of 30°. The direction that which individual rats exited the corner was recorded. The test was repeated ten times for each animal, and the percentage of right turns was calculated. The foot-fault test examined the placement dysfunction of forelimbs. Rats were put on an elevated grid surface and placed their paws on the wire while moving along the grid. The total number of steps and the number of foot-faults for the left forelimb the rat used to cross the grid were counted. Data were presented as the percentage of foot faults per the total number of steps.

### Hematoxylin and eosin (H&E) staining

H&E staining kit (Solarbio life science, Beijing, China) was used according to the manufacturer’s instructions. Briefly, sections were stained with hematoxylin staining solution for 10 min, Subsequently, washed with tap water for 3 min. Then, the brain sections were differentiated in 1% hydrochloric acid-ethanol for 30 s, rinsed in distilled water for 5 min, counterstained with eosin staining solution for 2 min, and dehydrated with 95% alcohol, Finally, cleared in xylene and covered with a coverslip. Then, the cellular morphology was observed under the microscope.

### Nissl’s staining

Nissl staining was performed to detect neuronal injury. After deparaffinization, sections were washed in PBS and incubated in Nissl’ staining solution (Beyotime Biotechnology, Shanghai, China) for about 5 min. Brain sections were then rinsed in PBS and dehydrated in gradient ethanol, cleared in xylene, and covered with a coverslip. Survival neurons were then observed under the microscope.

### Fluoro-Jade C staining

Fluoro-Jade C staining was applied to evaluate neurodegeneration. The sections were immersed in a solution of 1% sodium hydroxide in 80% alcohol for 5 min, 70% alcohol for 2 min, distilled water for 2 min, and followed by 0.06% potassium permanganate for 10 min with gently shaking. The sections were immersed in a solution of FJC (Millipore Corporation, United States) in acetic acid for 30 min. Then rinsed three times in distilled water and allowed to dry at 50°C for 15 min before covering and observing.

### Immunofluorescence

The paraffin section (5 μm-thick) of the brain was prepared, after being fixed, washed, permeabilized, and blocked, cells were incubated with rabbit anti-ZO-1 (1:200, Abcam), or rabbit anti-Iba-1 (1:100, Proteintech). After that, sections were further incubated with the fluorescein 488-conjugated anti-rabbit antibody (1:1000, Jackson Immunoresearch). Slides were imaged using a fluorescence microscope. To explore the polarization reprogramming effects of DEX, we performed immunofluorescence double labeling. Sections were incubated in mouse anti-Iba-1 (1:100, Abcam), with rabbit anti-iNOS (1:100, Abcam) or rabbit anti-CD206 (1:100, Abcam) for 1 h at 37°C. Antibodies were then used: the fluorescein 488-conjugated anti-rabbit antibody (1:1000, Jackson Immunoresearch, United States), or Cy3-conjugated anti-mouse antibody (1:1000, Jackson Immunoresearch).

### TdT-mediated dUTP nick end labeling staining

Paraffin-embedded brain sections were deparaffinized and rehydrated. To assess cellular apoptosis, the sections were incubated with a proteinase K working solution (15 μg/mL in 10 mM Tris/HCl, pH 7.5) for 30 min at 37°C. After washing three times with PBS buffer, they were then incubated with 50 μL of TUNEL reaction mixture, covered with a lid, and incubated for 2 h at 37°C in the dark. Then, the slides were incubated with 100 μL stopping buffer for 10 min and then rinsed in PBS three times. DAPI was applied to detect the nuclei. Images were observed *via* a fluorescence microscope, and the percentage of the dUTP-positive cells was detected.

### Western blot analysis

For western blot analysis, tissues adjacent to the hematoma were obtained. It was performed as described previously (Zhou et al., 2018). The following primary antibodies were used: rabbit anti-β-actin (1:6000, Abcam), or rabbit anti-Bax (1:500, Servicebio), or rabbit anti-Bcl-2 (1:1000, Proteintech), or rabbit anti-ZO-1 (1:1000, Proteintech), or rabbit anti-occludin (1:500, ABclonal), or mouse anti-TNF-α (1:1000, Proteintech), or mouse anti-IL-1β (1:1000, Abcam), or rabbit anti-IL-10 (1:700, Abcam), or rabbit anti-TGF-β (1:800, Proteintech), or rabbit anti-claudin5 (1:1000, Abcam), or rabbit anti-Iba-1 (1:1000, Cell Signaling Technology), or rabbit anti-p-IκBα (1:1000, Cell Signaling Technology) or mouse anti-IκBα (1:1000, Cell Signaling Technology). The immunopositive bands were visualized using an enhanced chemiluminescent substrate (Thermo Fisher, Waltham, MA, United States) and Bio-Rad ChemiDoc XRS digital documentation system. The amount of protein expression is presented relative to the levels of β-actin. The nuclear/cytoplasmic proteins in fresh brain tissues were isolated using the Nuclear/Cytoplasmic Fractionation Kit (Beyotime Biotechnology, Shanghai, China). Nuclear/cytoplasmic proteins were used for NF-κB p65 detection; PVDF membranes were probed with primary or rabbit anti-NF-κB p65 (1:1000, Cell Signaling Technology), rabbit anti-β-actin (1:6000, Abcam), or mouse anti-Histone H3 (1:5000, Cell Signaling Technology). The amount of cytoplasmic NF-κB p65 is presented relative to the levels of β-actin; nuclear NF-κB p65 is shown relative to the stories of Histone H3.

### Evans blue extravasation

Evans blue (100 mg/kg) was injected into the tail vein. After 1 h, the rats were perfused with saline. The brain tissues around the hematoma were collected. After weighing, brain tissue was placed in a 50% trichloroacetic acid solution. Then, the tissues were homogenized and centrifuged (12,000 rotations/min, 20 min). The Supernatants were collected and analyzed at 620 nm using a spectrometer. EB leakage was quantified by a standard curve and was normalized by EB/brain (μg/g).

### Brain water content measurement

The brains of the animals were removed and weighed (wet weight). Then the brains were dried at 105°C for 24 h, and the weight of the samples was measured (dry weight). Brain water content was defined as (wet weight-dry weight)/wet weight × 100%.

### Statistical analysis

All data were expressed as the mean ± SD. Repeated measures ANOVA (RM-ANOVA) was employed for behavioral tests. The remaining data were analyzed by using the student *t*-test and one-way ANOVA. The criterion for statistical significance was *p* < 0.05.

## Results

### Dexmedetomidine exerted neuroprotective effects against intracerebral hemorrhage-induced neurological deficits

**FIGURE 1:**
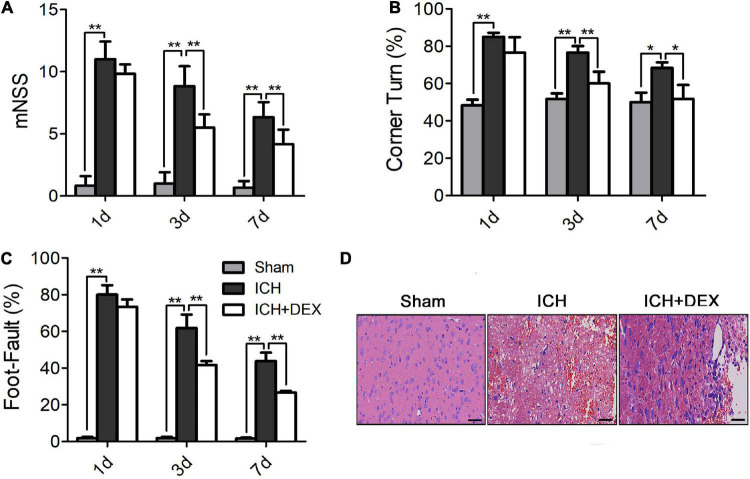
**(A)** The DEX markedly ameliorated total neurological deficit scores and improved neurological functions compared to the ICH group (a decrease in the mNSS) on days 3 and 7 after ICH. Additionally, the DEX-treated rat significantly outperformed the ICH rat in the corner turn test **(B)** and the foot-fault test **(C)** (on day3 and 7). **(D)** On the 3rd day post-ICH, the neuronal necrosis and degeneration in the DEX-treated group were less serious than in the ICH group. Scale bar = 50 μm. Values are expressed as mean ± SD. *p < 0.05, **p < 0.01.

The results of the mNSS testing showed that the neurological status of the ICH group and DEX-treated group improved over time. As shown in Figure 1A, the DEX markedly ameliorated total neurological deficit scores and improved neurological functions compared to the ICH group (a decrease in the mNSS) on days 3 and 7 after ICH. ICH induction significantly increased the corner turn test compared with sham animals on days 1, 3, and 7 (Figure 1B). The corner turn test was significantly ameliorated by the administration of DEX (Figure 1B). Additionally, we conducted a foot-fault test to compare the sensorimotor behavior of the rats in the different treatment groups with that of the rats in the ICH group on days 1, 3, and 7. The rats in the ICH group showed a significantly increased number of foot faults compared with the rats in the sham group from day 1 to day 7 (Figure 1C). However, treatment with DEX reduced the number of foot faults compared with the ICH group from day 3 to day 7 (Figure 1C).

### Dexmedetomidine alleviated neuronal cell loss and apoptosis in intracerebral hemorrhage

**FIGURE 2:**
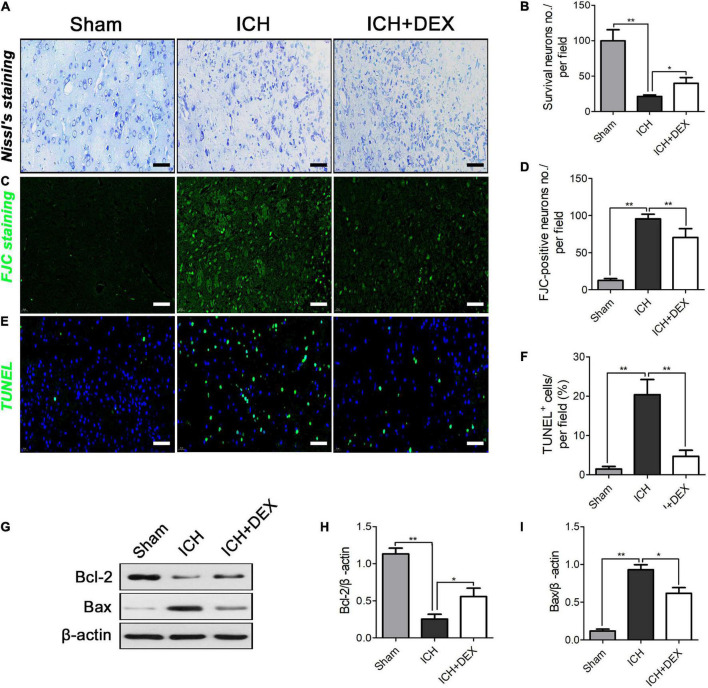
**(A,B)** Compared with the Sham group, the number of surviving neurons were significantly reduced after ICH. However, after being administered with DEX, more surviving neurons were observed three days post-ICH. On the 3rd day post-ICH, **(C,D)** a significantly increased number of FJC-positive cells was observed in the ICH group compared with the Sham group. However, DEX treatment markedly reduced the number of FJC-positive cells. **(E,F)** ICH could induce obvious neuronal cell loss and apoptosis in the rat brain. DEX could dramatically decrease the ratio of TUNEL^+^ cells in ICH (3rd-day post-ICH). **(G)** Representative Western blot expressions of Bcl-2 and Bax **(H,I)** Bax were markedly diminished by DEX. While, compared with the ICH group, Bcl-2 was significantly increased on the 3rd day post-ICH.*p < 0.05, **p < 0.01. Scale bar = 50 μm.

On the 3rd-day post-ICH, the morphology was observed by HE staining. The outcomes showed that the morphology of the sham group was the round and intact nucleus. Severe nuclear concentration, loose staining, and cell death could be found around the hematoma of the ICH group. The pathological degree of the DEX-therapy group in the corresponding region was prominently reduced contrasted the ICH group (Figure 1D). Subsequently, we performed Nissl’s staining to evaluate neuron viability in the perihematomal area of ICH. Compared with the sham group, the number of surviving neurons was significantly reduced after ICH (Figures 2A,B). However, after being administered with DEX, much more surviving neurons were observed (Figures 2A,B). FJC was used to evaluate the severity of neuronal degeneration (Figures 2C,D). The FJC-positive cells were observed considerably increased in the ICH group compared with the sham group. Nevertheless, the DEX treatment group markedly alleviated the number of FJC-positive cells after ICH (Figures 2C,D). To further detect neuronal cell loss and apoptosis, a TUNEL assay was conducted. ICH could induce apparent neuronal cell loss and apoptosis in the rat brain (Figures 2E,F). DEX could dramatically diminish the ratio of TUNEL^+^ staining cells (Figures 2E,F). Western blot analysis demonstrated that treatment with DEX prevented the ICH-induced Bax expression (Figures 2G,I). Moreover, DEX enhanced the anti-apoptotic Bcl-2 protein expression (Figures 2G,H).

### Dexmedetomidine treatment protected blood-brain barrier integrity of intracerebral hemorrhage rat

**FIGURE 3:**
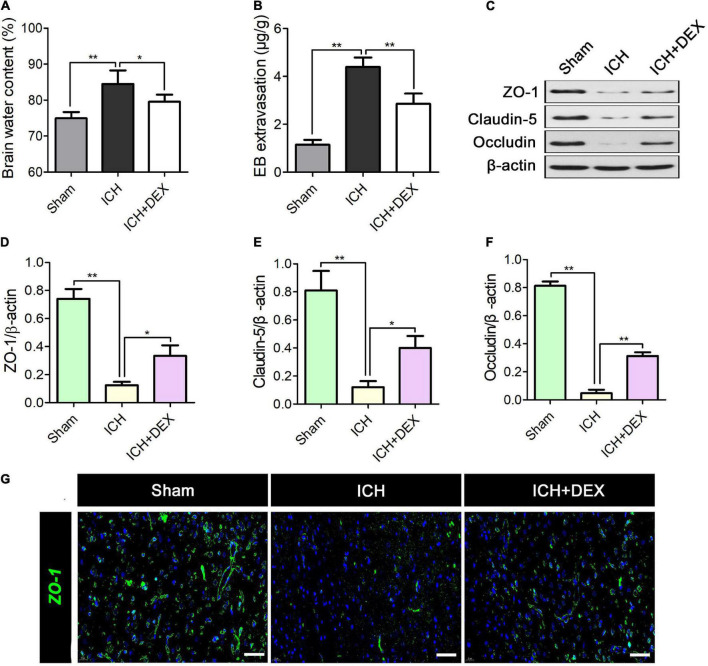
On the 3rd-day post-ICH, **(A)** quantitative analysis of brain water content and **(B)** Evan’s blue analysis. **(C)** Representative Western blot expressions of ZO-1, occludin, and claudin-5. **(D–F)** Reduced levels of ZO-1, occludin, and claudin-5 was observed in the ICH group compared with the Sham group. However, the levels of the ZO-1, occludin, and claudin-5 increased in DEX group. **(G)** Immunofluorescence analysis showed similar results for the expression of ZO-1. *p < 0.05, **p < 0.01. Scale bar = 50 μm.

On the 3rd-day post-ICH, Brain water content and EB extravasation were observed to assess the BBB integrity. As shown in Figure 3A, ICH remarkably enhanced the brain water content. In contrast, DEX administration dramatically reduces brain water content at 3rd after ICH (Figure 3A). Additionally, EB extravasation was elevated in the ICH group compared with the sham group (Figure 3B). Yet the rats receiving the DEX displayed a lower level of EB leakage (Figure 3B). Accumulating evidence demonstrated that the restrictive nature of the BBB is precisely due to tight junctions between adjacent endothelial cells. Hence, the tight junction proteins including ZO-1, occludin, and claudin-5 were also detected. Figures 3C–F showed that reduced ZO-1, occludin, and claudin-5 were observed in the ICH group compared with the sham group. However, the levels of the ZO-1, occludin, and claudin-5 increased in DEX group. Immunofluorescence analysis which observed the perihematomal area showed similar results of the expression of ZO-1 (Figure 3G).

### Dexmedetomidine suppressed microglia/macrophage activation but enhanced M2 microglia/macrophage polarization in the intracerebral hemorrhage brain

**FIGURE 4:**
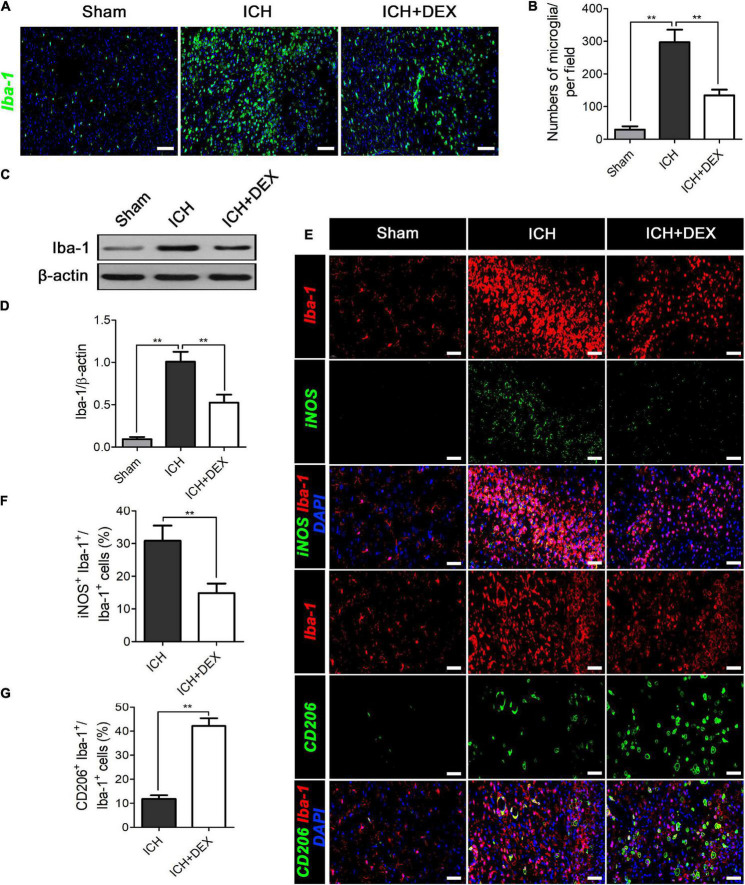
On the 3rd-day post-ICH, **(A,B)** immunofluorescence revealed the DEX group significantly reduced the microglia/macrophage expression around hematomas compared with the ICH group. Scale bar = 100 μm. **(C)** Representative Western blot expressions of Iba-1. **(D)** The expression of the Iba-1 protein was significantly upregulated in the ICH compared with the Sham group. However, treatment with DEX could notably suppress activated microglia/macrophage levels. **(E,F)** DEX reduced the ratio of Iba-1^+^ cells that co-expressed with M1-associated marker-iNOS. **(E,G)** The Iba-1^+^ cells that co-expressed with M2-associated marker-CD206 significantly increased in the DEX-treated group. **p < 0.01. Scale bar = 50 μm.

Microglia/macrophages are the primary immune cells in the central nervous system and play a non-negligible role in response to ICH-induced damages. The actions of DEX on microglia/macrophage in ICH were explored on the 3rd-day post-ICH. The results of western blot analysis revealed that the expression of Iba-1 (a marker for microglia/macrophage activation) protein was significantly upregulated in the ICH compared with the sham group (Figures 4C,D). However, treatment with DEX could notably suppress the activation of microglia/macrophage (Figures 4C,D). Similarly, immunofluorescence revealed the DEX group significantly reduced the activated microglia/macrophage expression around hematomas compared with the ICH group (Figures 4A,B).

After ICH, microglia/macrophages exhibit dynamic polarization over time. We further determined the role of DEX in the modulatory effects of triggering the phenotypic conversion of microglia/macrophages, double immunofluorescent staining was examined around the hematoma of all groups. The ratio of Iba-1^+^ cells that co-expressed with M1-associated marker-iNOS was reduced by treatment of DEX (Figures 4E,F). At the same time, the percentage of Iba-1 positive cells that co-expressed with M2-associated marker-CD206 was significantly increased in the DEX-treated group (Figures 4E,G).

### Dexmedetomidine significantly diminished pro-inflammatory cytokines expression, and simultaneously promoted anti-inflammatory cytokines expression

**FIGURE 5:**
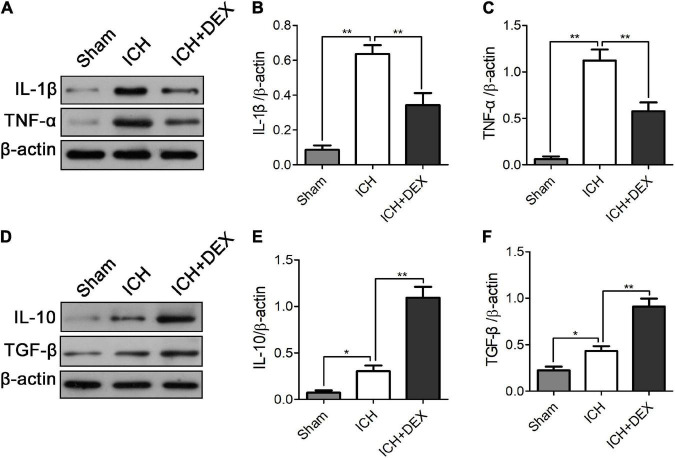
On the 3rd-day post-ICH, compared with the sham operation group, the protein levels of M1 expression products, including TNF-α and IL-1β, and M2 expression products, including IL-10 and TGF-β, were significantly increased in the ICH group. DEX significantly decreased the production of TNF-α and IL-1β **(A–C)**. Simultaneously, enhanced IL-10 and TGF-β were observed in the DEX group **(D–F)**. *p < 0.05, **p < 0.01.

M1-like microglia/macrophages mainly express proinflammatory cytokines, while M2-like microglia/macrophages mainly express anti-inflammatory cytokines. An uncontrolled and prolonged M1-activated state in ICH contributes to additional neuronal damage. Compared with the sham operation group, the protein levels of M1 expression products, including TNF-α and IL-1β, and M2 expression products, including IL-10 and TGF-β, were significantly increased in the ICH group on day 3. DEX significantly decreased the production of TNF-α and IL-1β (Figures 5A–C). Simultaneously, enhanced IL-10 and TGF-β were observed in the DEX group (Figures 5D–F).

### Dexmedetomidine inhibited nuclear translocation of NF-κB in intracerebral hemorrhage rats

**FIGURE 6:**
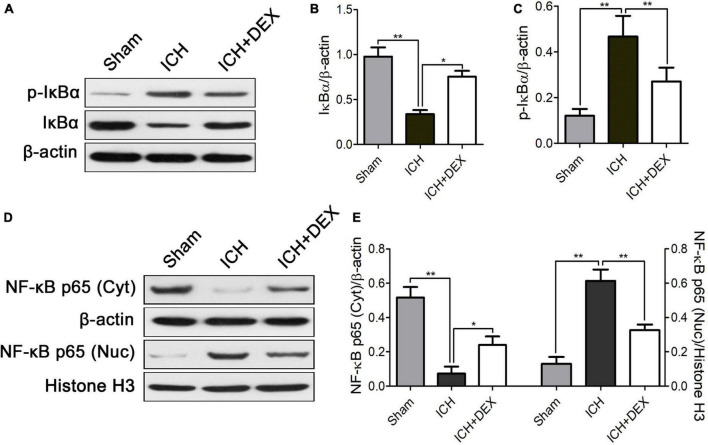
On the 3rd-day post-ICH, **(A)** representative Western blot expressions of IκBα and p-IκBα. **(B,C)** The protein expression of IκBα was significantly decreased with an increase in p-IκBα in the ICH group compared with those of the sham group. DEX caused marked dephosphorylation of IκBα. **(D)** Representative Western blot expressions of NF-κB. **(E)** Translocation of NF-κB p65 into the nucleus occurred after ICH. DEX significantly blocked the translocation. *p < 0.05, **p < 0.01. Scale bar = 50 μm.

The NF-κB signaling pathway is a crucial regulator of inflammatory responses and microglia/macrophage activation. Therefore, for underlying mechanism exploration, we detected the NF-κB signaling pathway in the brain of the ICH model. The protein expression of IκBα was significantly decreased with an increase in p-IκBα in the ICH group compared with those of the sham group (Figures 6A–C). DEX caused marked dephosphorylation of IκBα (Figures 6A–C). Moreover, translocation of NF-κB p65 into the nucleus occurred after ICH (Figures 6D,E). DEX significantly blocked the translocation.

## Discussion

The significant findings of the current study are as follows: (1) DEX promoted the integrity remodeling of BBB, which plays a protective role in the ICH brain, (2) It indicated that DEX post-conditioning induced the microglia/macrophages polarization reprogramming after ICH, (3) Moreover, the underlying mechanism attributed to the inhibiting of NF-κB signaling pathway. To the best of our knowledge, this is the first study to describe a beneficial effect of DEX on BBB integrity-related microglia/macrophage polarization after ICH.

The BBB is formed by cerebral endothelial cells and their linking tight junctions. It has been demonstrated that BBB can limit the passive diffusion of compounds from the blood to the brain. Following ICH, the disruption of BBB integrity can be triggered by the release of cytokines and chemokines the production of reactive oxygen species (ROS), and subsequent oxidative stress (Zeng et al., 2017; Guo et al., 2020). In animals, it has been reported that there is a noticeable increase in BBB permeability (Takagi et al., 2017). The increased BBB permeability facilitates the infiltration of leukocytes and inflammatory mediators into the perihematomal tissue, which may cause secondary brain injury in ICH. Maintaining the integrity of the BBB is crucial in ICH. Tight junction proteins including ZO-1, claudin-5, and occludin regulate BBB paracellular permeability (Lohmann et al., 2004; Jiao et al., 2011; Yang et al., 2019). Under normal conditions, tight junctions are composed of transmembrane proteins that undergo homomeric interactions on adjacent endothelial cells to seclude the paracellular pathway. However, after ICH, the loss of these tight junction proteins from the plasma membrane occurs, which will increase BBB permeability and further destruct its integrity (Gindorf et al., 2021). In the current study, DEX remarkably improved the water content and Evan’s blue extravasation of the ICH brain and simultaneously increased ZO-1, claudin-5, and occludin proteins. This phenomenon coincided with improved neurological functions, which confirmed the brain-protective effects of DEX.

Microglia/macrophage is the primary immune cell in the central nervous system (CNS). Accumulating evidence revealed that reactive microglia/macrophages likely contributed to the functional changes of BBB after ICH (Chen and Trapp, 2016; Chen et al., 2019). Once activated, microglia change from a ramified, quiescent morphology lacking endocytotic and phagocytotic activity to an ameboid, activated morphology. It plays a dual role. The M1 microglia/macrophages (classically activated), which involve the production and secretion of reactive oxygen species and proinflammatory cytokines (such as TNF-α, IL-1β, and IL-6), are typically assumed to promote brain damage (Higashi et al., 2017; Yu et al., 2019). Whereas, the M2 phenotype (alternatively activated), which exerts functions of phagocytosis, removes damaged cell debris, secretes neurotrophic factors, and anti-inflammatory mediators (such as IL-10, IL-4, and TGF-β), is believed to possess neuroprotective properties (Akhtar et al., 2017; Calvello et al., 2017). The strategy in which therapeutics promote M2 microglia/macrophage polarization is shown to preserve functional BBB integrity of the damaged brain (Yang et al., 2015; Tschoe et al., 2020). Iba-1 is a well-established protein marker of microglia/macrophages that is selectively upregulated and inactivated (Hamzei Taj et al., 2016). In Figure 4C, immunofluorescence analysis showed that more Iba-1 positive cells could be observed after ICH, and morphologically the cells appeared to be ameboid. It indicated the activated state of the microglia/macrophage. Further results showed the M1 phenotype inhibiting and simultaneously M2 microglia/macrophage polarization promoting effects of DEX. The microglia/macrophage polarization reprogramming role may contribute to its protective role in ICH.

An NF-κB signaling pathway plays crucial role in the activation of the microglia/macrophages by controlling the transcription of associated genes (Park et al., 2015). Phosphorylation and degradation of the IκBα protein promote the activation of NF-κB (Jing et al., 2015; Oguiza et al., 2015; Yang et al., 2020). Here, we found that DEX significantly attenuated the increase in phosphorylation of IκBα in ICH rats. In addition, it also reversed the IκBα degradation. Previous studies also found that activated NF-κB regulates inflammatory response and BBB integrity after ICH (Liu et al., 2014; Zeng et al., 2017; Wen et al., 2018; Zhu et al., 2018). Following ICH, multiple factors, such as thrombin, TNF-α, and IL-1β, can induce IκB phosphorylation and NF-κB activation (Denes et al., 2011; Zhang et al., 2014, 2015b; Yin et al., 2017). Previous research also proved that activated NF-κB regulates the expression of TNF-α and IL-1β (Zhang et al., 2015a; Su et al., 2017). Inhibition of the IκBα protein phosphorylation has an important inhibitory effect on NF-κB protein activation. It further causes an important inhibitory effect on the expression of the downstream inflammatory factors TNF-α, and IL-1β. In the present study, compared with the sham operation group, the protein levels of TNF-α and IL-1β were significantly increased in the ICH group on day 3. DEX significantly decreased the production of TNF-α and IL-1β (Figures 5A–C). Human and animal models of ICH experiments indicated that there was a close relationship between NF-κB activation and cell death (Bhakar et al., 2002; Ridder and Schwaninger, 2009; Zhang et al., 2014, 2015b). Bcl-2 has been proven to promote neuronal regeneration and survival and have anti-apoptotic effects (Yuan et al., 2014; Shen et al., 2016; Bobinger et al., 2018), while Bax has an apoptosis-promoting effect (Sun et al., 2017; Choi et al., 2018; Shen et al., 2018; Xu et al., 2019). In this study, cell death and apoptosis-related factors Bcl-2 and Bax were detected by western blotting to verify the effect of our intervention. Western blot analysis demonstrated that treatment with DEX prevented the ICH-induced Bax expression (Figures 2G,I). Moreover, DEX enhanced the anti-apoptotic Bcl-2 protein expression (Figures 2G,H).

In conclusion, our data demonstrate that DEX post-conditioning in ICH facilitates the microglia/macrophage phenotype switch from pro-inflammatory (M1 phenotype) to anti-inflammatory (M2 phenotype), which contributes to the DEX-mediated neuroprotection in ICH. The therapeutic effect depends on the suppression of the NF-κB signaling pathway.

## Data availability statement

The original contributions presented in this study are included in the article/Supplementary material, further inquiries can be directed to the corresponding authors.

## Ethics statement

The animal study was reviewed and approved by the ethics committee of Shanxi Provincial People’s Hospital.

## Author contributions

JZ, ZY, and HG designed the experiments. HG wrote the manuscript. WZ, ZW, and HG performed the experiments. HG and ZL analyzed the data and visualized the figures. All authors contributed to the article and approved the submitted version.
