# Supplementary figures and images for: Dexmedetomidine post-conditioning protects blood-brain barrier integrity by modulating microglia/macrophage polarization via inhibiting NF-κB signaling pathway in intracerebral hemorrhage
Source: Front Mol Neurosci. 2022 Sep 8;15:977941. doi: 10.3389/fnmol.2022.977941 (PMC9512049; doi:10.3389/fnmol.2022.977941)

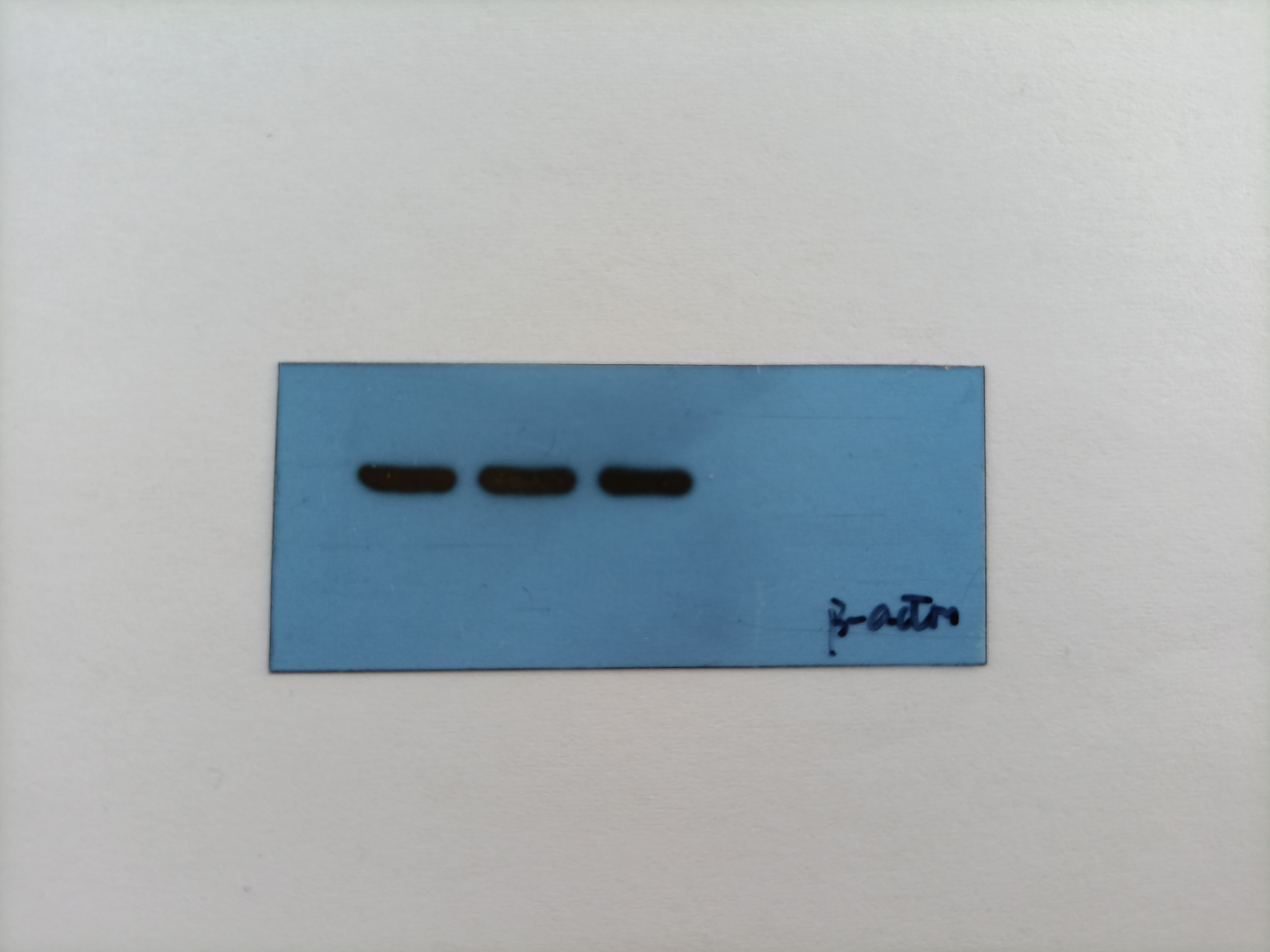

Supplement: Supplementary file 1 [file Image_1.jpg]

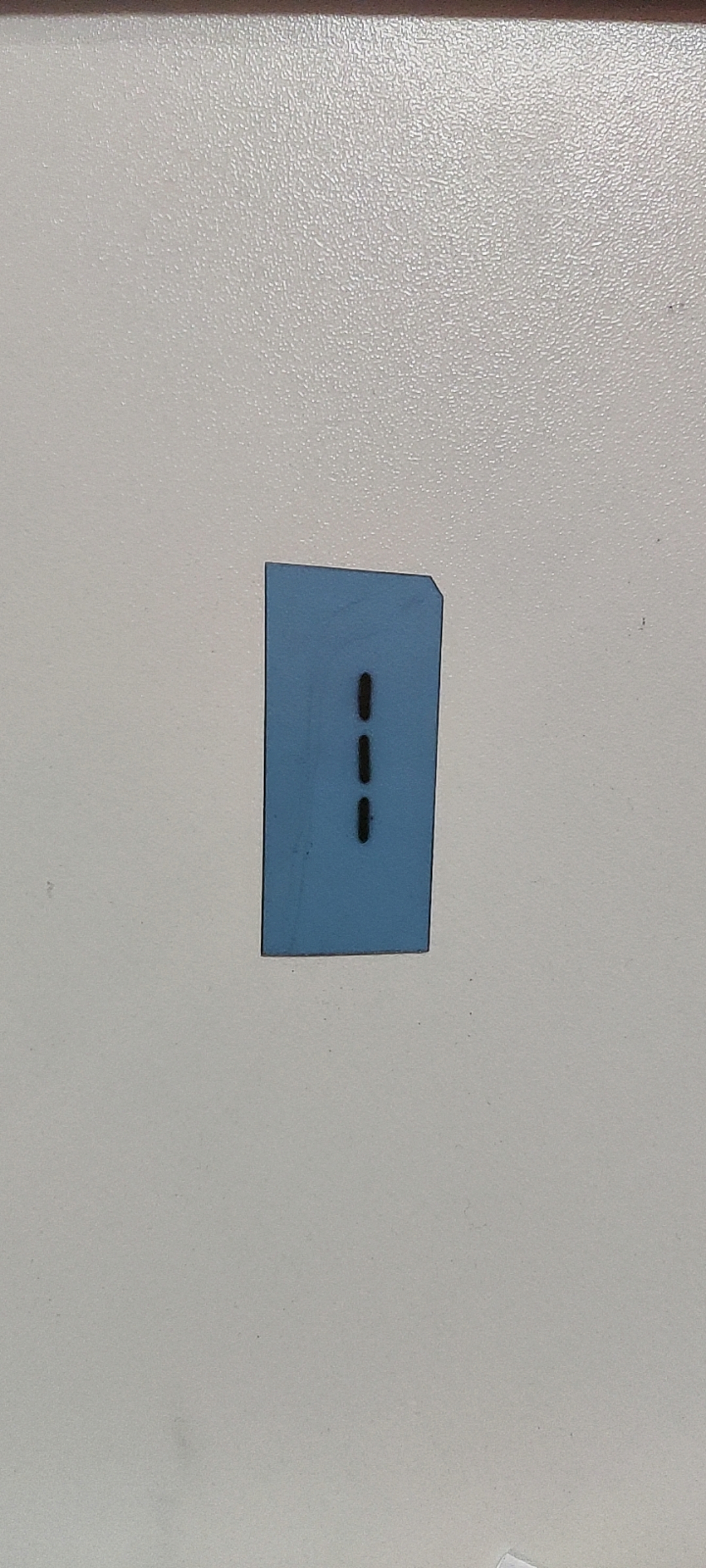

Supplement: Supplementary file 2 [file Image_2.JPEG]

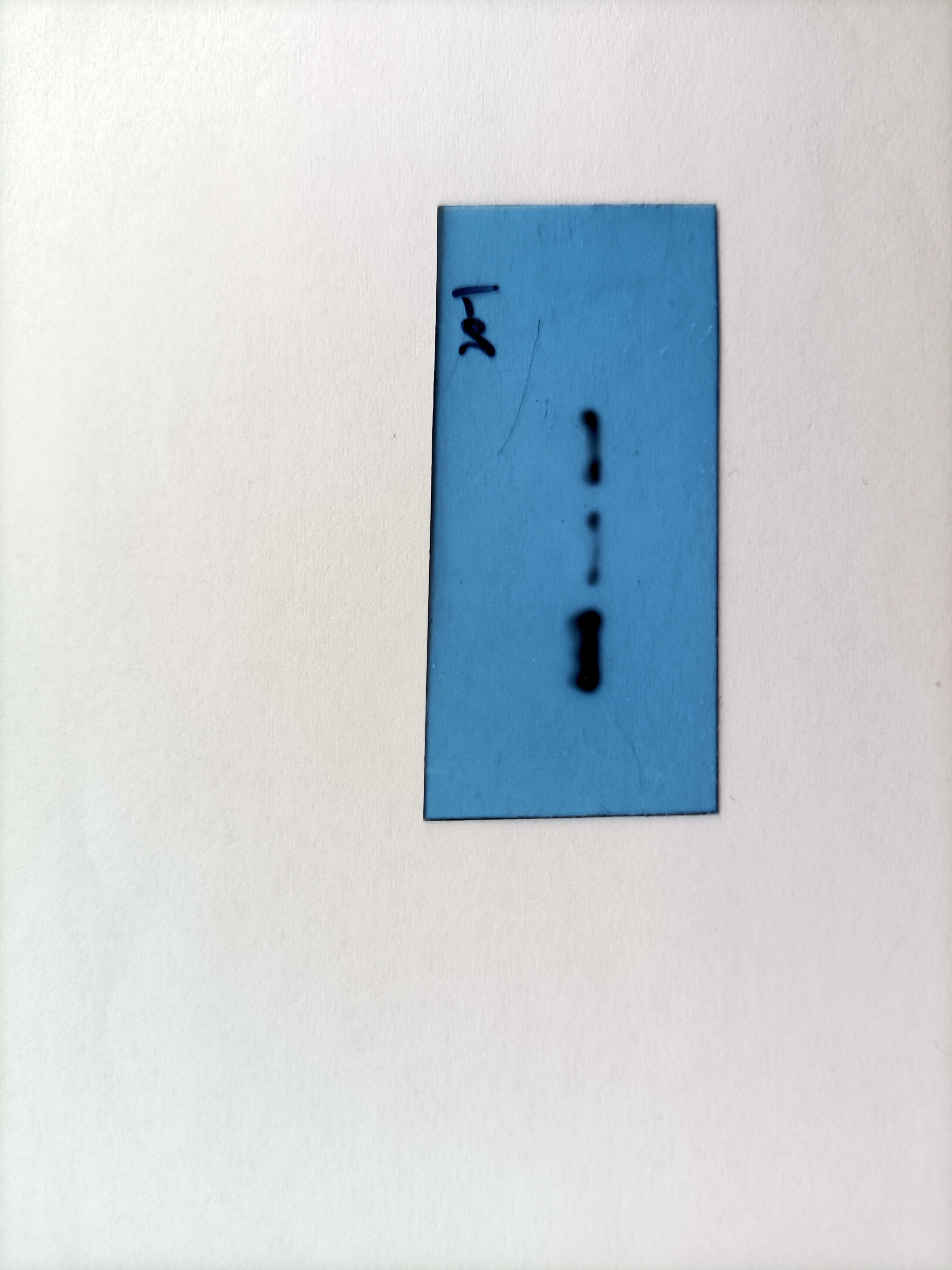

Supplement: Supplementary file 3 [file Image_3.jpg]

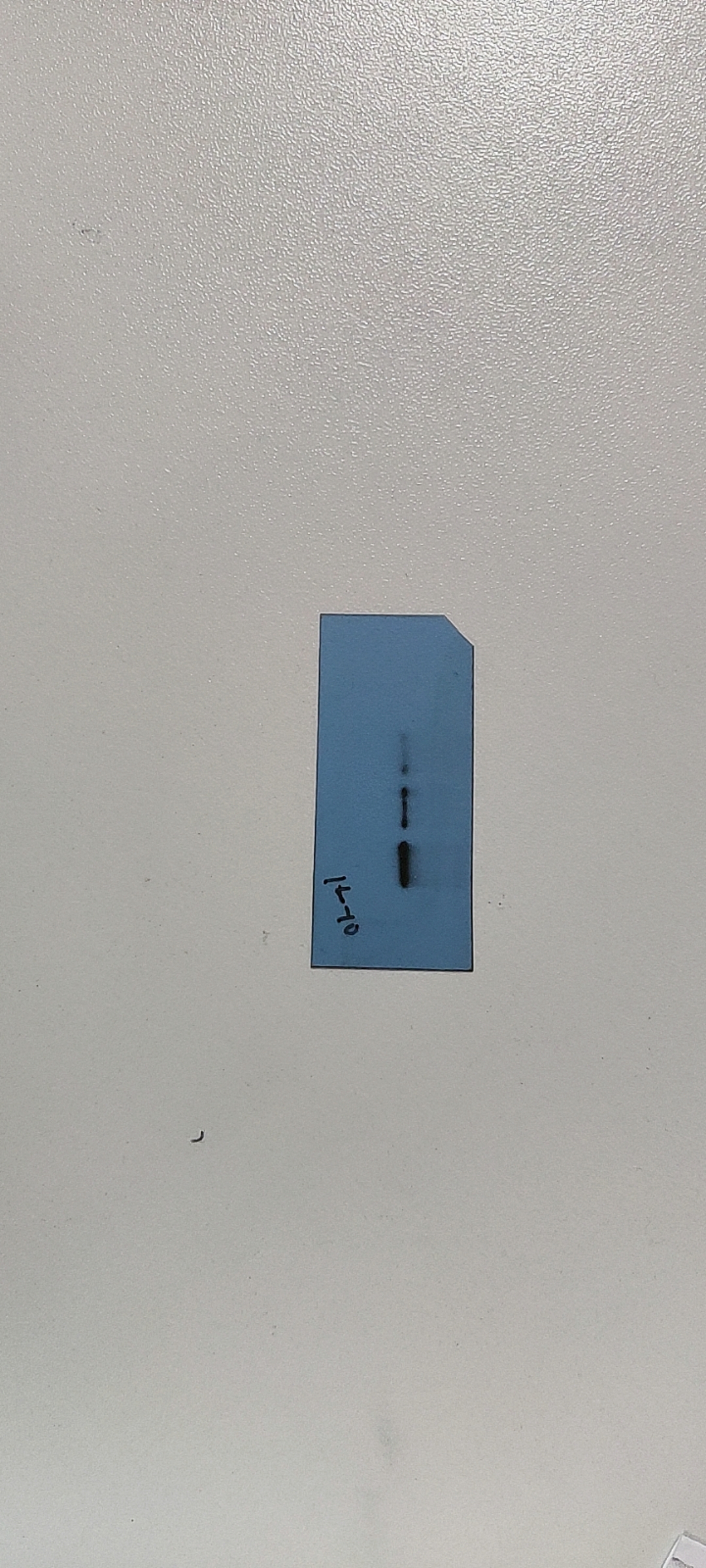

Supplement: Supplementary file 4 [file Image_4.JPEG]

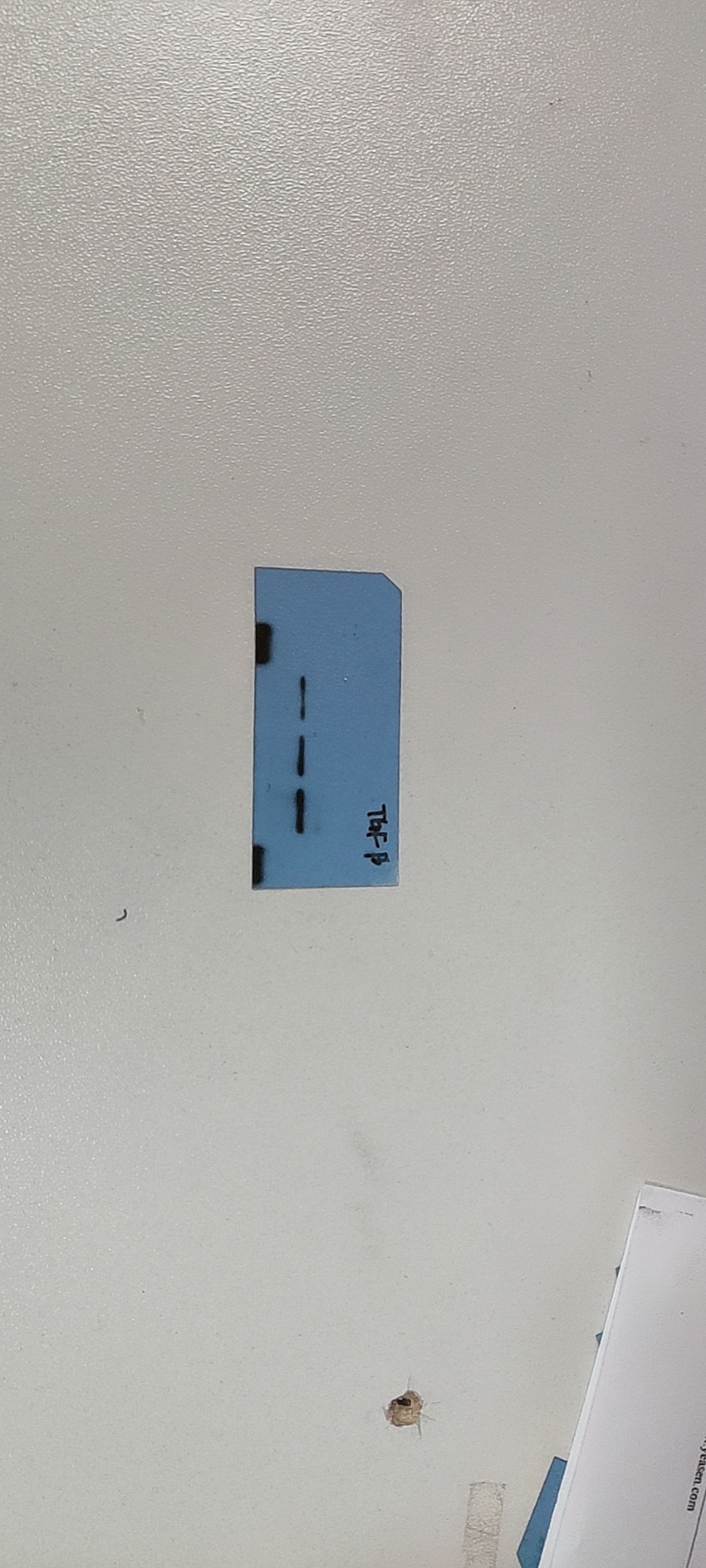

Supplement: Supplementary file 5 [file Image_5.JPEG]

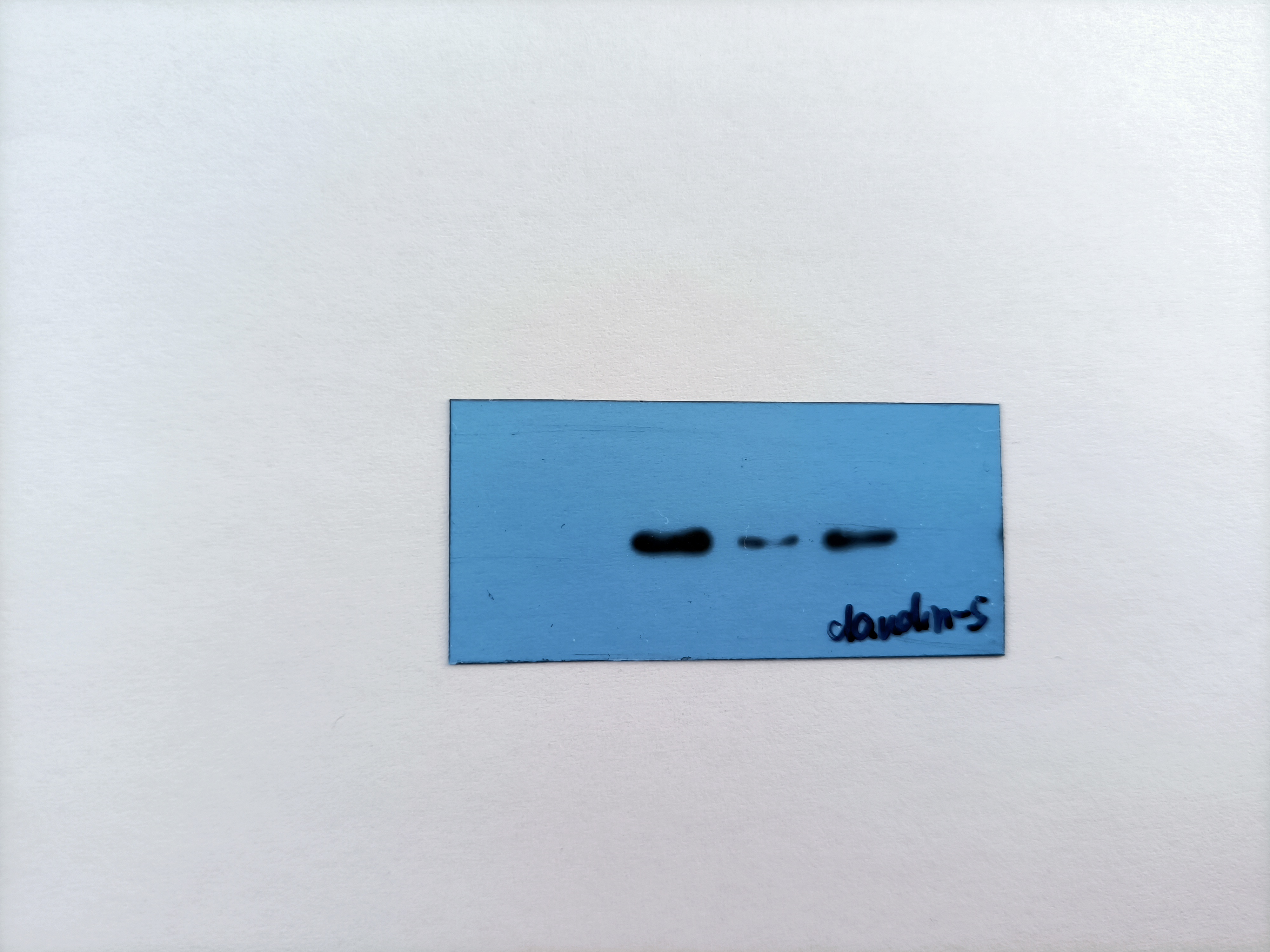

Supplement: Supplementary file 6 [file Image_6.jpg]

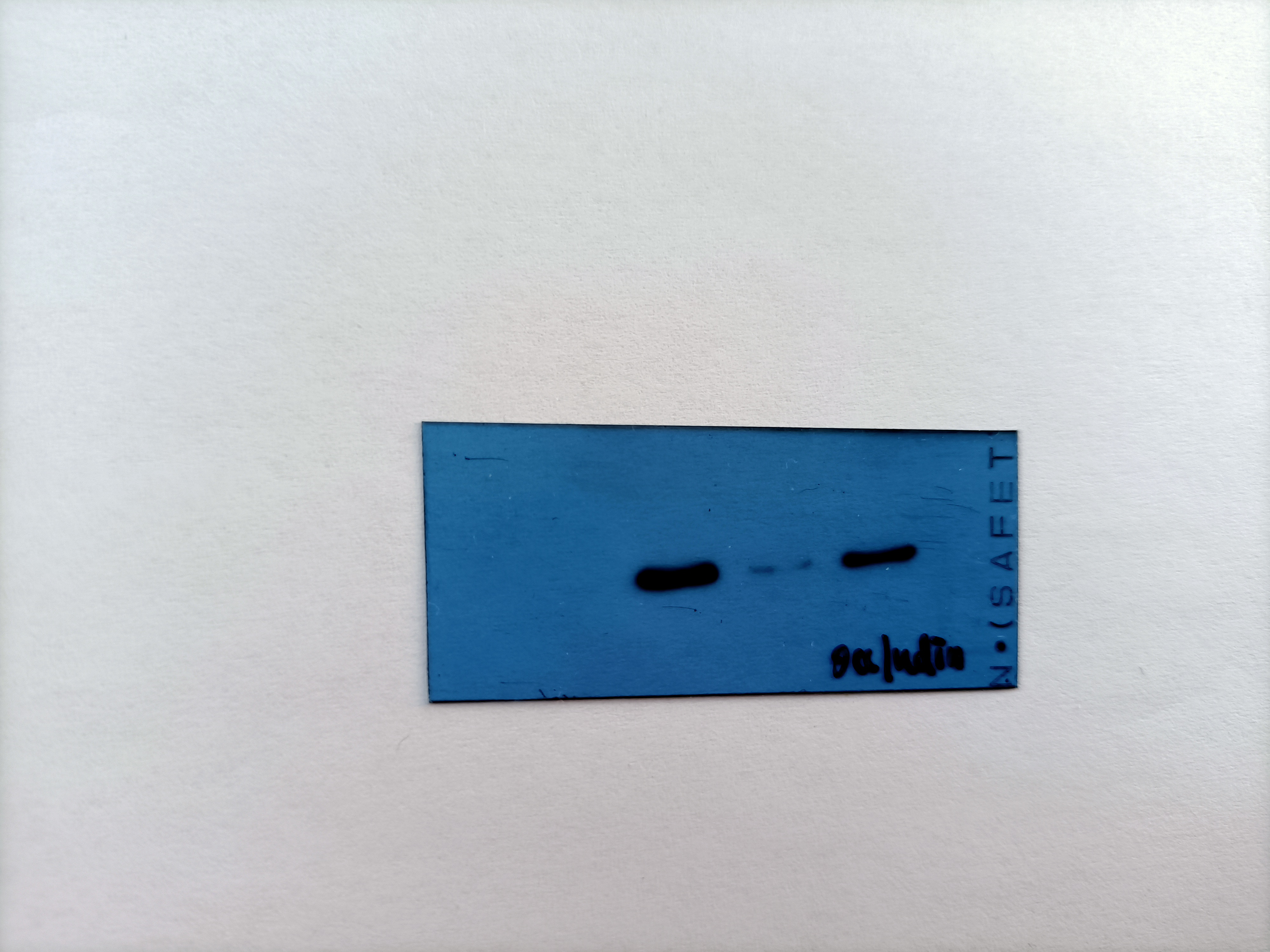

Supplement: Supplementary file 7 [file Image_7.jpg]

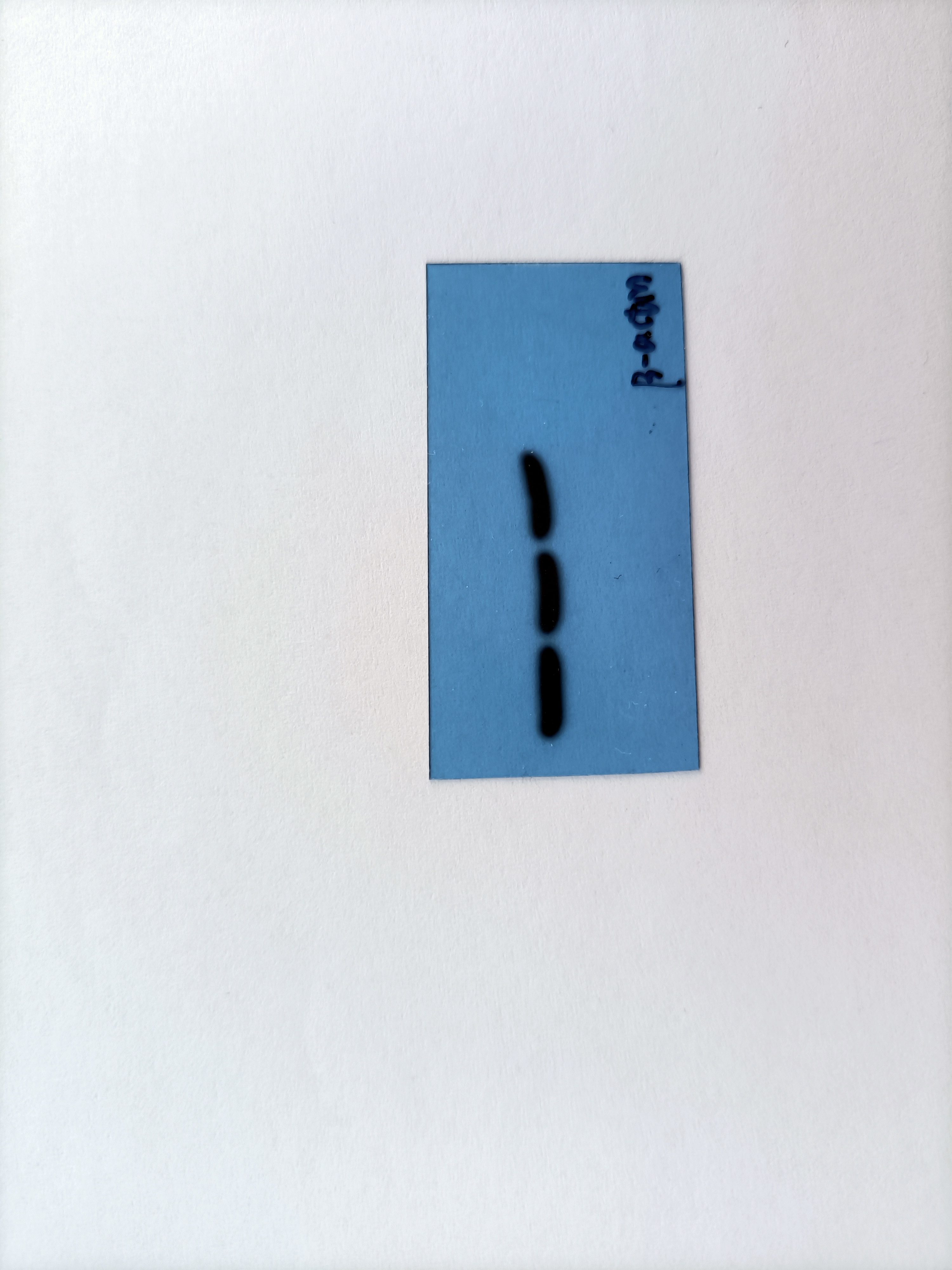

Supplement: Supplementary file 8 [file Image_8.jpg]

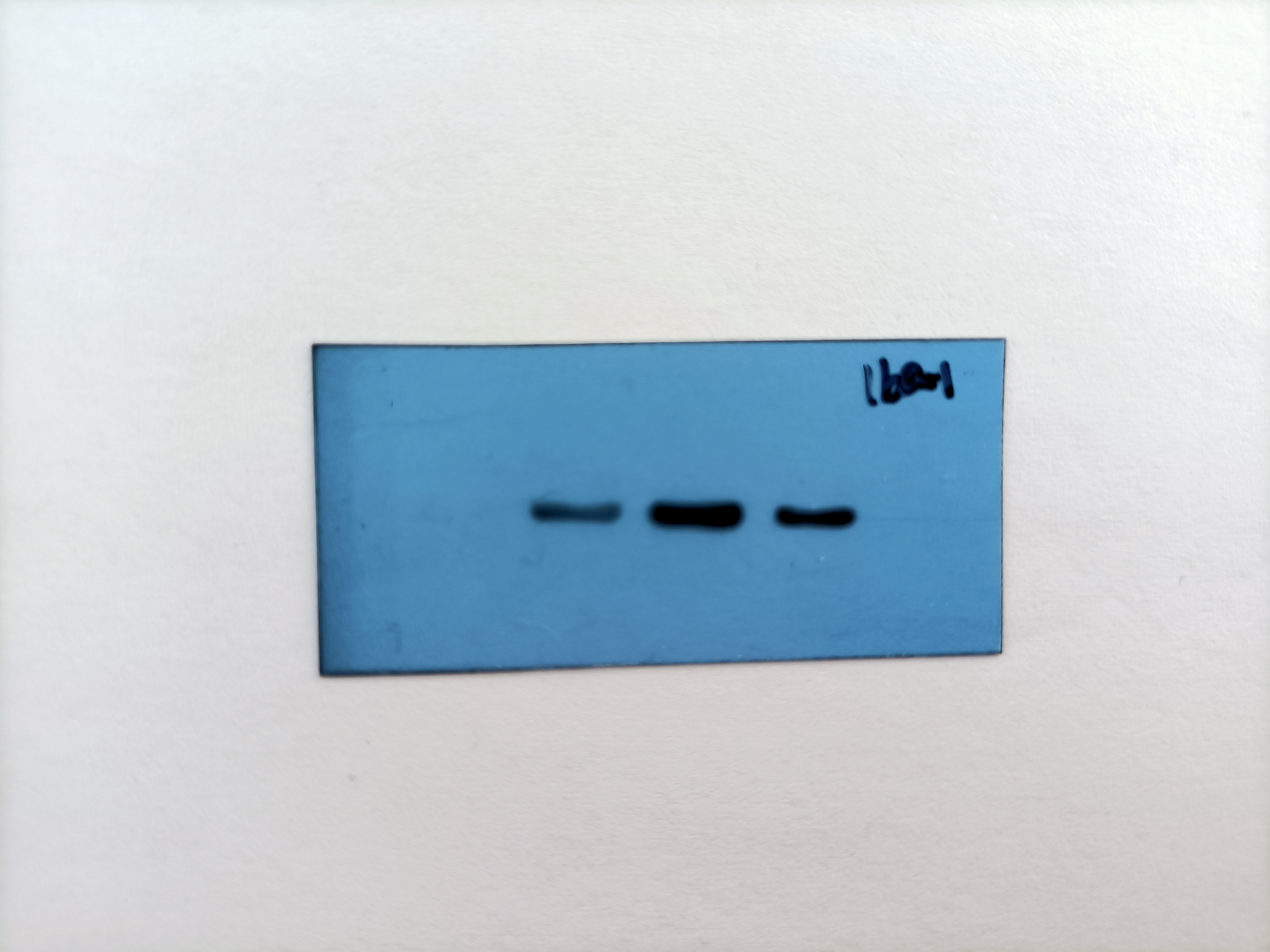

Supplement: Supplementary file 9 [file Image_9.jpg]
